# Regulation of *hlh-2* transcription during specification of the anchor cell of the *C. elegans* hermaphrodite gonad

**DOI:** 10.1093/g3journal/jkaf288

**Published:** 2025-12-02

**Authors:** Jee Hun Kim, Iva Greenwald

**Affiliations:** Department of Biological Sciences, Columbia University, 1212 Amsterdam Avenue, New York, NY 10027, United States; Department of Biological Sciences, Columbia University, 1212 Amsterdam Avenue, New York, NY 10027, United States

**Keywords:** *hlh-2*, gonad, lateral specification, transcription, *C. elegans*, anchor cell, *lin-12*

## Abstract

The anchor cell (AC) of the *Caenorhabditis elegans* hermaphrodite somatic gonad primordium is a signaling nexus that regulates uterine and vulval development. As the somatic gonad primordium is forming, four cells, two α and two β cells, are born with the potential to be the AC. This potential becomes restricted to the α cells, which undergo the LIN-12/Notch-mediated AC/VU decision to resolve which α cell will become the AC. The transcription factor HLH-2, the sole E/Daughterless protein ortholog in *C. elegans*, is critical for this process, and dynamic regulation of *hlh-2* transcription contributes to the robust specification of a single AC. The *hlh-2*prox regulatory element mediates the dynamic pattern of *hlh-2* transcription: the initial expression in the parents of the α and β cells, which is briefly sustained in the α and β cells after they are born; its subsequent restriction to the α cells during the AC/VU decision; and its continued expression in the AC. In this study, we identify the cis-acting sequences within *hlh-2*prox and transcription factors required for the initial expression of *hlh-2* in the α and β cells and their parents, demonstrate that the Wnt/β-catenin Asymmetry Pathway (WβA) regulates restriction of *hlh-2* transcription to the α cells, and show that the maintenance of *hlh-2* transcription in α cells requires distinct elements and the chromatin factor LSY-12. This analysis extends our understanding of regulatory mechanisms that operate during a precise and robust Notch-mediated lateral specification paradigm.

## Introduction

**Fig. 1.**
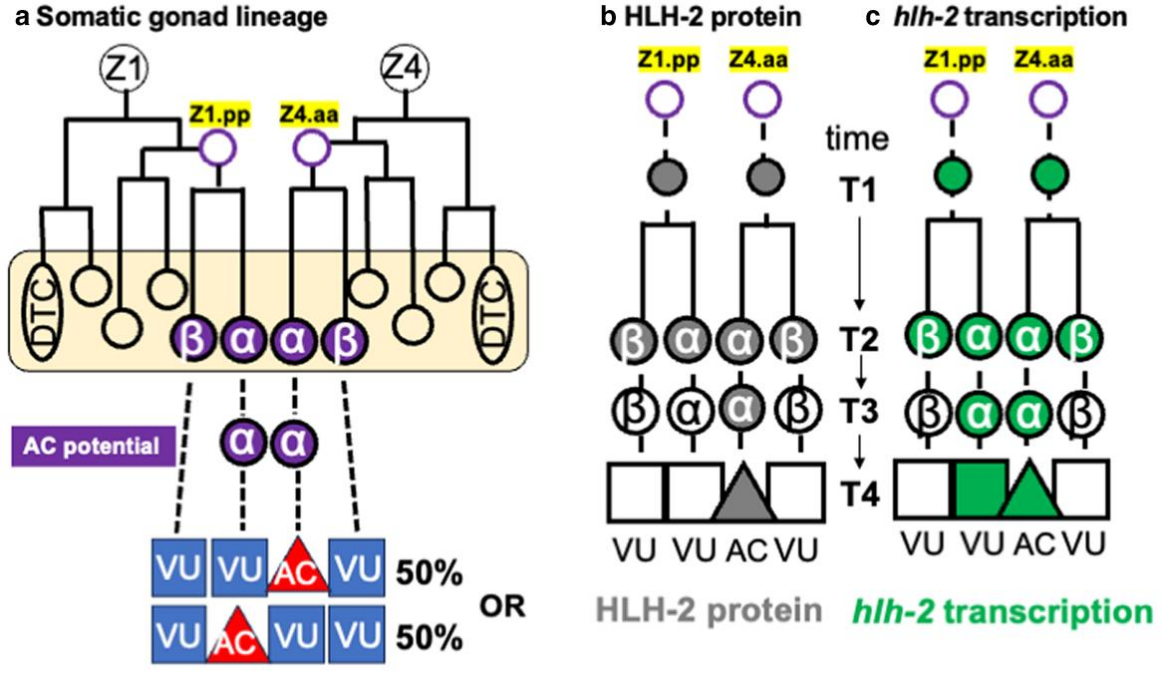
Early somatic gonad lineage, featuring *hlh-2* transcriptional reporter and HLH-2 protein expression, in wild-type *C. elegans* hermaphrodites. a) Somatic gonad lineage through somatic primordium formation. The hermaphrodite somatic gonad descends from the progenitor cells Z1 and Z4 by mirror-symmetric lineages. The daughters of Z1.pp and Z4.aa, the α and β cells, are initially born with the potential for AC fate. The distal β cells (Z1.ppa and Z4.aap) invariably become VUs, while the proximal α cells maintain AC potential until the outcome of the AC/VU decision. In 50% of animals, the Z1-descended α cell (Z1.ppp) will become the AC (with Z4.aaa becoming a VU), and in the other 50%, the Z4-descended α cell (Z4.aaa) will become the AC (with Z1.ppp becoming a VU). Stochastic events lead one parent, Z1.pp or Z4.aa, to express HLH-2 first, endowing one of the two α cells with an edge in *lin-12* activity that is amplified by LIN-12/Notch-mediated signaling between them to resolve which will be the AC and which the VU. b) HLH-2 protein. Endogenously-tagged or transgenically-expressed GFP::HLH-2 is initially present in the α and β cells but is degraded in response to LIN-12 activation so it is restricted to the AC. c) *hlh-2* transcription. *hlh-2* is initially transcribed in the α and β cells and their parents. Expression of various transcriptional reporters that include *hlh-2*prox, including the *hlh-2* transcriptional reporter shown in Fig. 3 (*arSi155*), reveal that a transcriptional difference between α and β cells persists after the AC and VU fates have been specified (see also Fig. 4a).

The *C. elegans* L1 hermaphrodite hatches with a four-celled gonad primordium that includes two somatic precursors, Z1 and Z4, which generate the cells that form the mature, two-armed gonad (Kimble and Hirsh 1979). Gonadogenesis occurs in two phases. The first phase spans the L1 and L2 larval stages and culminates in the generation of the somatic gonad primordium, which has a distal-proximal axis defined by a distal tip cell (DTC) at each distal end and the anchor cell (AC) at the center (proximal) (Fig. 1a). During the second phase of gonadogenesis, the DTCs lead gonadal arm outgrowth, the AC serves as a critical signaling hub that organizes uterine and vulval development, and the nine somatic gonad blast cells generate the structural cells of the sheath, spermatheca, and uterus.

As the somatic gonad primordium is forming, four cells are born with the potential to be the AC (Kimble 1981; Seydoux et al. 1990): Z1.ppp and Z4.aaa, also called α cells, and their sisters, Z1.ppa and Z4.aap, the β cells. The transcription factor HLH-2, the sole E/Daughterless protein ortholog in *C. elegans* (Krause et al. 1997), has multiple, sequential roles in this paradigm (Karp and Greenwald 2003, 2004). Initially, *hlh-2* endows the α and β cells with the potential to be the AC; the β cells rapidly lose the potential to be the AC and always become ventral uterine precursor cells (VUs), but the two α cells retain the potential to be the AC or a VU. LIN-12/Notch-mediated interactions between them resolves which α cell will be the AC and which will be a VU, a process called the “AC/VU decision” (Seydoux and Greenwald 1989; Greenwald 2012). Once the AC has been specified, *hlh-2* continues to be required for differentiated functions of the AC such as expressing the EGF-like ligand that induces the vulva (Hwang and Sternberg 2004). For all of these roles, HLH-2 functions as a homodimer (Sallee and Greenwald 2015).

The AC/VU decision is stochastic in that every hermaphrodite has just one AC, but in a population of hermaphrodites, the AC fate is assumed by Z1.ppp in half of the animals and by Z4.aaa in the other half [(Kimble and Hirsh 1979); Fig. 1a]. However, high-throughput lineage analysis demonstrated that endogenous GFP::HLH-2 expression, initially observed within a narrow window after the birth of a parent cell, is highly predictive of the outcome of the AC/VU decision: the first parent to express GFP::HLH-2 gives rise to the αVU. This “HLH-2 expression bias” along with the finding that the initial expression of *lin-12* requires *hlh-2* led to the proposal that the first parent cell to express HLH-2 gives its α daughter an “edge” in LIN-12 activation, thus biasing it to be the αVU (Attner et al. 2019). The predictions of this model have been supported by analysis of a biosensor for Notch activation (Shaffer and Greenwald 2022b).

The patterns of *hlh-2* transcription and HLH-2 protein stability correlate with its different roles in establishing AC potential and resolving the fates of the α cells (Fig. 1b and c) (Sallee and Greenwald 2015; Benavidez et al. 2022). Transcription of *hlh-2* begins in Z1.pp and Z4.aa, the parents of the α and β cells, and is initially sustained in the α and β cells, consistent with the role of HLH-2 in endowing these cells with AC potential. Then, transcription of *hlh-2* is lost in the β cells, consistent with the observation that β cells lose their AC potential soon after their birth; transcription of *hlh-2* is sustained in the α cells, consistent with the requirement for HLH-2 promoting transcription of *lag-2* as the α cells undergo the LIN-12-mediated AC/VU decision. Initially, HLH-2 protein correlates with transcription of *hlh-2* in the α cells, but during the AC/VU decision, LIN-12 activation leads to degradation of HLH-2, so the HLH-2 protein is degraded in the presumptive αVU and becomes restricted to the presumptive AC even as both α cells continue to report *hlh-2* transcription.

The *hlh-2*prox regulatory element mediates the dynamic expression pattern of *hlh-2* in the α and β cells and their parents (Sallee and Greenwald 2015; Attner et al. 2019). In this study, we investigated three features of transcriptional regulation mediated by *hlh-2prox*. First, we show that the initial expression of *hlh-2* in the parents of the α and β cells (hereafter, “the parents”), which will have a deterministic effect on the AC/VU decision later, is mediated by cis-acting regulatory sequences shared with *nhr-67*, another gene expressed in the α and β cells that contributes to the AC/VU decision (Verghese et al. 2011; Bodofsky et al. 2018; Medwig-Kinney et al. 2023). Using RNAi targeting candidate factors predicted to recognize these sequences, we identify AT-hook and bZIP transcription factors that appear to regulate early expression of both genes. Second, we provide strong support for the hypothesis that the loss of *hlh-2* transcription in β cells is mediated by the Wnt/β-catenin Asymmetry Pathway (WβA) by using RNAi and Auxin-Inducible Degradation to manipulate WβA components. Finally, we demonstrate that the maintenance of *hlh-2* transcription in α cells requires distinct α cell Maintenance Elements (αMEs) and the chromatin factor LSY-12.

## Materials and methods

### *Caenorhabditis elegans* genetics and developmental synchronization

See Supplementary Tables 1 and 2 for additional strain and allele details. Strains were maintained at 20 °C and temperature conditions during each experiment are indicated below. All injections were performed on healthy young adult hermaphrodites. Information about genes was derived from the gene page of several WormBase versions (Sternberg et al. 2024).

*hlh-2(ar623[gfp::hlh-2]) I* is a knock-in allele for endogenously-tagged GFP::HLH-2 (Attner et al. 2019).

*hlh-2(ar614) I* is a deletion within the 5′ regulatory element of *hlh-*2prox that is null for *hlh-2* in the proximal somatic gonad (Attner et al. 2019).

*lsy-12(ot170) V* and *lsy-12(ot171) V* are recessive alleles of *lsy-12* mapped to the final exon of the *lsy-12a* isoform that induce ASE neuronal and Pvul defects (Sarin et al. 2007; O'Meara et al. 2010).

*nhr-67(syb509[nhr-67::gfp]) IV* is a knock-in allele for endogenously-tagged NHR-67::GFP (Medwig-Kinney et al. 2023).

*nhr-67(pf88) IV* is a deletion in the 5′ regulatory region of *nhr-67* that induces 2AC and vulval defects (Verghese et al. 2011).

*nre-1(hd20)lin-15B (hd126) X* confers hypersensitivity to RNAi (Schmitz et al. 2007).

*pop-1(he335[egfp::pop-1) I* is a knock-in allele for endogenously-tagged GFP::POP-1 (van der Horst et al. 2019).

*arIs51[cdh-3::gfp] IV* was used as an AC marker (Karp and Greenwald 2003).

*arTi112[ckb-3p::mCherry::his-58::unc-54 3′UTR] V* and *arTi145[ckb-3p::mCherry::his-58::unc-54 3′UTR] II* were used to mark somatic gonad cells with mCherry (Attner et al. 2019).

*arTi237[ckb-3p::Cre(opti)::tbb-2 3′UTR] X* is a somatic gonad-specific Cre driver (Shaffer and Greenwald 2022a).

*arTi443[rps-27p::TIR1F79G9(flexon)::unc-54 3′UTR] V* is a *lox2272-*flexon-based (Shaffer and Greenwald 2022a) transgene which expresses TIR1^F79G^ (Hills-Muckey et al. 2022) in cells that express Cre (Wittes and Greenwald 2024).

L1 arrest was used to obtain developmentally-synchronous cultures after feeding. A standard bleaching protocol (Stiernagle 2006) was used to obtain eggs, which were pipetted into an Erlenmeyer flask containing 10 mL of M9 buffer to induce L1 arrest. After shaking for 24 to 36 h at 20 °C, arrested L1 larvae were placed onto NGM seeded with OP50 bacteria or “feeding RNAi” bacteria and grown at 25 °C for the appropriate time as indicated for each experiment below.

### CRISPR/Cas9-based single copy transcriptional reporters for *hlh-2*prox deletion analysis and EAE mutants in *hlh-2* and *nhr-67*

We used the pWZ111 backbone, which permits CRISPR/Cas9-mediated single copy insertion into the *ttTi4348 I* locus (Pani and Goldstein 2018). The plasmid inserting wild type *hlh-2* transcriptional reporter into *ttTi4348*, pHK42, was generated by the following method. The 5,252 bp region upstream of *hlh-2* cloned from N2 genomic DNA, codon-optimized GFP flanked by N-terminal SV40 and C-terminal *egl-13* nuclear localization sequences, and *unc-54* 3′ UTR were assembled into pWZ111 digested with NotI and AvrII using the Gibson assembly protocol (Gibson 2011). The version of *hlh-*2prox (326-bp) analyzed in this study for all experiments differs from the original 327-bp *hlh-*2prox sequence described in Sallee et al. (2015) in that the guanine at the −5253 position upstream of *hlh-2* ATG is not included, and thus is shorter by one base; the wild type GFP reporters generated (*arSi155, arSi214*) are expressed at high levels in α and β cells, their parents, and α cells following AC specification. The wild-type transcriptional reporter plasmid served as the template for generating transcriptional reporters of *hlh-2* with deletions and substitutions in the *hlh-2*prox sequence. Truncations and substitutions on the *hlh-*2prox sequence, for the deletion analysis and analysis of the EAEs, respectively, were cloned from this plasmid and then reassembled into pHK42 that had been digested with SpeI and AgeI, which removed the original wild type *hlh-2*prox, using the Gibson assembly protocol. EAE substitutions, which mutated the ATTGCGY sites into TTTTTTY, either replaced the first EAE only (5,126–5,121 > A, number indicates bp upstream of *hlh-2* ATG), the second EAE only (5,084–5,079 > A), or both of the EAEs (5,126–5,121 > A; 5,084–5,079 > A).

The 3,710 bp region upstream of *nhr-67* cloned from N2 genomic DNA, codon-optimized GFP flanked by N-terminal SV40 and C-terminal *egl-13* nuclear localization sequences, and *unc-54* 3′ UTR were assembled into pHK35 (a slightly modified version of pWZ111 where the ccdB cassette removed by NotI and AvrII was exchanged with GFP) digested with NotI and AvrII (which rendered the vector sequentially identical to pWZ111 digested with NotI and AvrII) using the Gibson assembly protocol (Gibson 2011) to generate a wild type transcriptional reporter plasmid of *nhr-67* (pHK68). To make the EAE mutant transcriptional reporter plasmid of *nhr-67* (pHK69), we inserted a long oligo with the EAE substitution mutations (1,706–1,701 > A; 1,642–1,637 > A, number indicates bp upstream of *nhr-67* ATG), mutating the ATTGCGY sites into AAAAAAY, and fragments of the *nhr-67* 3,710 bp upstream region cloned from pHK68 into pHK35 digested with NotI and AvrII. We note that 1,797 bp upstream of *nhr-67* ATG, there is an 18 bp-long cytosine repeat in the reference genome which is 17 bp long in both pHK68 and pHK69.

All plasmids were sequenced for verification. The transgenes described above were inserted into *ttTi4348* in N2 animals using CRISPR/Cas9 (Pani and Goldstein 2018).

### CRISPR/Cas9-based endogenous mutations

We mutated the endogenous EAE sites of *hlh-2* and *nhr-67* and inserted an AID tag at the 3′ end of *lit-1* using the protocol as described by (Ghanta and Mello 2020). The repair templates for *hlh-2* and *nhr-67* were cloned as PCR fragments from pHK62 and pHK69, respectively. The repair template for *lit-1* consisted of a flexible linker and the AID degron sequence flanked by small homology arms for repairing the Cas9 cut site on *lit-1* which was cloned from a pBluescript-based plasmid with AID degron flanked by flexible linkers (pHK57). The crRNAs were ordered from IDT and consisted of the following: *nhr-67*:/AltR1/rCrGrC rArArU rArUrC rGrArG rArArC rGrArU rGrCrG rUrUrU rUrArG rArGrC rUrArU rGrCrU/AltR2/; *hlh-2*:/AltR1/rArUrG rArArU rArArC rUrCrA rUrUrG rCrGrC rArArG rUrUrU rUrArG rArGrC rUrArU rGrCrU/AltR2/; *lit-1*:/AltR1/rUrUrU rUrUrU rUrGrU rCrArC rCrArA rGrCrC rUrGrG rUrUrU rUrArG rArGrC rUrArU rGrCrU/AltR2/. The injection mix for *hlh-2* was injected into GS9222 [*hlh-2(ar623[gfp::hlh-2]) I*; *arTi112V*] to generate GS10134 [*hlh-2(ar623ar668[hlh-2p(Δboth EAE)::gfp::hlh-2]) I*; *arTi112V]*, the injection mix for *nhr-67* was injected into PHX509 [*nhr-67(syb509[nhr-67::gfp]) IV*] (Medwig-Kinney et al. 2023) to generate *nhr-67(syb509ar667[nhr-67p(Δboth EAE)::nhr-67::gfp]) IV,* and the injection mix for *lit-1* was injected into N2 animals to generate *lit-1(ar665[lit-1::AID]) III*. Homozygosed strains were verified by genotyping and sequencing.

### CRISPR/Cas9-based knock-in reporter

We created wrmScarlet::HLH-2 using the protocol from (Dickinson et al. 2015). The self-excising cassette (SEC) repair template pYR1, containing the *hlh-2* homology arms and wrmScarlet, and the Cas9-sgRNA construct plasmid pJB56, which targeted the sequence “AGTTTTCAGAACCTCAATGG”, was generated by a former graduate student Justin Benavidez. The generated allele *hlh-2(ar657[wrmScarlet::hlh-2]) I* expresses the wrmScarlet tag as expected including in the AC, VUs, distal tip cells, sex myoblasts, and head neurons. The worms are healthy and lay embryos copiously. There is an extra “A” insertion 18 bp upstream of the ATG, which likely occurred during the repair as it is adjacent to the sgRNA sequence.

### MiniMos-based transgenes

All plasmids were generated with Gibson assembly (Gibson 2011) with backbone derived from the MiniMos vector (pCFJ910) and the *unc-54 3′UTR* and were sequenced for verification. The transgenes were injected into N2 animals following protocol from Frøkjær-Jensen et al. (2014). Single-copy random insertions were mapped following standard protocol (Frøkjær-Jensen et al. 2014).

pJB78[*ckb-3p::mTagBFP2::his-11::unc-54 3′UTR*] was used to generate a single-copy mTagBFP2-based somatic gonad reporter allele *arTi448 II*.

pHK23[*hlh-2p(5.2 kb)::tdTomato(2xnls)::unc-54 3′UTR*] was used to generate *arTi460 III,* a single-copy reporter of *hlh-2* transcription in tdTomato flanked by N-terminal SV40 and C-terminal *egl-13* nuclear localization sequences. A clone of the 5.2 kb 5′ regulatory sequence of *hlh-2* (Sallee and Greenwald 2015) was assembled with *tdTomato(2xnls)* and *unc-54* 3′ UTR.

pHK56[*rps-27p::mCherry(flexon)::his-58::unc-54 3′UTR*] was used to generate *arTi481 I* and *arTi483 III, loxP-*flexon-based single-copy somatic gonad cell markers. The first intron of mCherry was replaced with a flexon (Shaffer and Greenwald 2022a) which prevents expression of mCherry by the strong ubiquitous promoter *rps-27p* until the flexon has been excised by Cre. The combination of this transgene with *arTi237*[*ckb-3p::Cre(Opti)::tbb-2 3′UTR] X* gives strong expression in somatic gonad cells.

### Sequence analysis

The *Caenorhabditis elegans hlh-*2prox sequence used in this study (−5,252 to −4,927) and intergenic sequences upstream of *hlh-2* in *C. remanei*, *C. briggsae*, and *C. japonica*, and *hlh-2.1* and *hlh-2.2* for *C. brenneri*, were obtained from the Wormbase genome browser (https://wormbase.org) and ClustalW (Thompson et al. 1994); https://www.genome.jp/tools-bin/clustalw and MAFFT (Katoh and Standley 2013; Katoh et al. 2019) https://mafft.cbrc.jp/alignment/server/ were used to align them. Following the criteria from Bodofsky et al. (2018), we sought to identify sites of common homology with 6 or more base pairs, but we did not identify any.

### Microscopy

For experiments that required quantification or were anticipated as potentially requiring quantification, we used a spinning disc confocal microscope to capture images with a single or dual camera system. Larvae were mounted on 4% agarose pads and immobilized with 10 mM levamisole. The following lasers were used for each fluorescent protein used in the study: 488 nm 100 mW laser for GFP, 561 nm 75 mW laser for mCherry and tdTomato, and 405 nm 50 mW laser for mTagBFP2. All images were captured with slices at intervals of 0.25 µm. Exposure times and laser power parameters for each experiment are described below. *Fiji* (Schindelin et al. 2012) was used for image analysis and *GraphPad Prism* (10.2.3, last updated July 2, 2024) was used for statistical analysis.

For other experiments, we used the 63x Plan-Apo objective lens inserted into a Zeiss Axio Imager D1 microscope, with X-Cite 120Q as the light source. Larvae were mounted on 3% agarose pads and immobilized with 10 mM levamisole.

### Deletion analysis of *hlh-2*prox

Strains GS10032, GS10001, GS10021, GS10033, GS10020, GS10031, GS10065, GS10068, and GS10060 were used for this analysis. In wild type, *hlh-2* is initially transcribed in all four α and β cells (T2) and is decreased in β cells over time while being maintained in α cells (T3). To capture this dynamic, larvae were imaged at 16 h after release from L1 arrest for T2, when *hlh-2* reporter is robustly expressed in all four α and β cells, and at 18.5 h for T3, when expression is relatively diminished in β cells. GFP expression was captured using the confocal microscope's single camera at 100 ms exposure time at 10% laser power. We used *Fiji* (Schindelin et al. 2012) to perform image analyses. For each α and β nucleus, GFP expression was used to draw the segmentation boundary. The top three slices for each cell in terms of integrated density were used to create sum *z*-projections, from which the integrated density was measured for analysis. Background was corrected by subtracting the integrated density of an area with the same dimension as the original segmentation boundary outside the larva from the raw integrated density.

### Initial expression of NHR-67::GFP in *hlh-2(ar614)* and GFP::HLH-2 in *nhr-67(RNAi)* and *nhr-67(pf88)*

Strain GS9640 was used for NHR-67::GFP expression analysis. Arrested L1 larvae were grown at 25 °C for 15 h, when the larvae were imaged for GFP expression using the confocal microscope's single camera at exposure of 300 ms at 15% laser power. The images were visually assessed for expression of NHR-67::GFP in the α and β cells and their parents. *nhr-67(RNAi)* was performed on GS8995 L1 larvae using the same protocol as described for the RNAi screen below. The RNAi clone was obtained from the Ahringer Library (Kamath et al. 2003). Larvae were scored 20.5 h later after release from L1 arrest for GFP::HLH-2 expression using the Zeiss Axio Imager D1 microscope. For GS10157, L2 animals were selected from a mixed-age plate grown at 20 °C and staged for gonadal development using the marker *ckb-3p::mCherry::H2B*; using the Zeiss Axio Imager D1 microscope, worms at the 8-cell stage were scored for presence of GFP::HLH-2 expression in the parents of α and β cells, and worms at the 12-cell stage were scored for presence of GFP:::HLH-2 in α and β cells.

### Analysis of *hlh-2* and *nhr-67* EAEs

#### Assessing EAE mutations in parents of α and β cells in single-copy insertion transcriptional reporters

Strains GS10032, GS10079, GS10113, and GS10115 were grown at 25 °C for 13 h after L1 arrest, around the timepoint when the parents of α and β cells divide. They were then imaged using the confocal microscope's camera at 200 ms at 10% laser power for *hlh-2p::GFP(2xnls)* and *nhr-67p::GFP(2xnls)* expression. Parents were identified based on morphology and whether the division into α and β cells had already occurred. Images of each larva were assessed in terms of whether GFP expression in a parent cell was visible in the proximal somatic gonad.

#### Assessing EAE mutations in α and β cells in single-copy insertion transcriptional reporters

Strains GS10079, GS10080, GS10089, GS10113, and GS10115 were grown at 25 °C after L1 arrest and imaged using the confocal microscope's camera at 100 ms at 10% laser power for *hlh-2p::GFP(2xnls)* and *nhr-67p::GFP(2xnls)* expression at T2 (16 additional hours of growth) and T3 (18.5 additional hours of growth). Given that α and β cells in images of GS10079 and GS10115 did not show visible expression in the vast majority of the samples, their images were assessed and compared with wild type in terms of whether GFP was visible in the α and β cells. The GFP expression in GS10080 and GS10089 was universally visible and was quantified with *Fiji* (Schindelin et al. 2012) as for the deletion analysis of *hlh-2*prox described above.

#### Assessing endogenous EAE mutations in parents of α and β cells

Strains GS9222, GS10133, GS10134, and GS10147 were grown at 25 °C for 13 h. They were then imaged using the confocal microscope's dual camera for *ckb-3p::mCherry::H2B* at exposure time of 800 ms at 30% laser power and their respective endogenously tagged GFP alleles at exposure time of 250 ms at 10% laser power. For each image, the parents of α and β cells were identified by *ckb-3p::mCherry::H2B* expression and then assessed for visible GFP expression.

#### Assessing the effect of endogenous EAE mutations after the birth of the α and β cells

For GS10133 and GS10147, L2 animals were selected from a mixed-age plate grown at 20 °C and staged for gonadal development using the marker *ckb-3p::mCherry::H2B;* worms at the 12-cell stage were scored for NHR-67::GFP expression in α and β cells with the Zeiss Axio Imager D1 microscope.

For GS10180 and GS10181, L3 animals were selected from a mixed-age plate grown at 20 °C and staged for gonadal development at the somatic gonad primordium stage based on gonad size; larvae were scored for visible wrmScarlet::HLH-2 expression in the α and β cells.

Given the transient nature of GFP::HLH-2 expression in α and β cells that become VUs in wild type animals, GFP::HLH-2 expression was scored in growth-synchronized worms for GS9222 and GS10134. Strains GS9222 and GS10134 were grown on NGM plates seeded with OP50 bacteria at 25 °C for 13.5 h after L1 arrest. L2 animals were staged for gonadal development using the marker *ckb-3p::mCherry::H2B;* worms at the 12-cell stage were scored for GFP::HLH-2 expression in in α and β cells with the Zeiss Axio Imager D1 microscope. To examine L4 phenotypes, L4 larvae of GS9222 and GS10134 were selected from a mixed-age plate grown at 20 °C and examined for whether vulval invagination had occurred with the Zeiss Axio Imager D1 microscope.

### RNAi screen for *trans* acting genes on *hlh-2* and *nhr-67* transcription

#### CIS-BP database

The documentation for the CIS-BP database is provided in Weirauch et al. (2014). We used the “Scan single sequences for TF binding” tool set to species “*C. elegans*” and motif model “PWMs—LogOdds” with threshold set at “8,” which was the default setting. We scanned upstream of sequences of *hlh-2* and *nhr-67* that contained their EAEs as well as adjacent 10 bases.

#### RNAi library

MacNeil et al. (2015) had constructed a comprehensive and annotated feeding RNAi library of 891 out of 934 predicted *C. elegans* transcription factors. We used an updated version of the RNAi library in which some clones were corrected, and annotations of the predicted DNA binding domains were used to identify genes that were predicted to have AT-hooks or to be C/EBP bZIP orthologs. We sequenced all clones before testing. RNAi plasmids for *attf-5*, *lsy-12*, and *pqn-75* were generated and transformed into HT115  *E. coli* cells by cloning exon-containing DNA fragments from N2 genomic DNA and inserting them into pGC480 (Korta et al. 2012) digested in NotI and HindIII following standard Gibson protocol (Gibson 2011). Additionally, the clone for *rad-26* was not correct so a correct clone was obtained from the Ahringer RNAi library (Kamath et al. 2003).

#### Preparation of worms

L1-arrested hermaphrodites from strains GS10123 or GS10160 were placed onto RNAi feeding plates seeded with a feeding RNAi strain, and each round had an empty vector L4440 plate as negative control. Plates were placed at 25 °C and scored at 20.5–21 h for “T2” and 24.5–25 h for “T3” (GS10123) and “T3/4” (GS10160). The *nre-1 (hd20) lin-15B (hd126)* background causes slower growth, hence the different timing from other experiments.

#### Scoring

Twenty worms were scored for each round, except for one round for the L4440 negative control for GS10123 in which only 17 larvae at the T2 stage could be found on the slide. We first scored depletion using the *hlh-2p::GFP(2xnls)* strain (GS10123). For any RNAi clone that yielded ≥5/20 worms defective in expression in either T2 or T3/4, we obtained triple replicates for GS10123 and also for GS10160 [*nhr-67p::GFP(2xnls)*].

Based on paralog analysis, (Rual et al. 2007) predicted that in *C. elegans*, RNAi off-target effects occur in mRNA that shares more than 95% identity over 40 nucleotides with dsDNA. BLAST analysis (wormbase.org) indicated that the RNAi sequences used in triple replicate experiments did not identify any nontarget genes that met this criterion.

#### Scoring for a change in GFP::HLH-2 expression by candidates from the RNAi screen

Candidates from the transcriptional reporter screen that were tested in replicates were depleted by RNAi for effect on endogenously-tagged GFP::HLH-2 expression. Screening protocol followed Benavidez et al. (2022). L1-arrested hermaphrodites from strain GS8995 were placed onto RNAi feeding plates seeded with a feeding RNAi strain; an empty vector L4440 plate as negative control and a *lin-12(RNAi)* plate as a positive control. Plates were placed at 25 °C and scored at 26  h for T3/4.

### Comparison of GFP::POP-1 and *hlh-2p::tdTomato(2xnls)* levels

L1 arrested hermaphrodites from strains GS9697 and GS9911 were placed onto NGM plates seeded with OP50 bacteria and left to grow in 25 °C for 16.5 h.

#### Imaging GFP::POP-1, *hlh-2*p::tdTomato, and somatic gonad markers

For GS9911, GFP::POP-1, tdTomato(2xnls), and mtagBFP2::H2B were imaged on the confocal microscope with a single camera with the following settings, respectively: 800 ms at 30% laser power, 800 ms at 30% laser power, and 1,250 ms at 40% laser power. In the strain GS9697, GFP::POP-1, and mCherry::H2B were imaged on the confocal microscope with dual camera with the following settings respectively: 800 ms at 30% laser power and 700 ms at 20% laser power.

#### Quantification of GFP::POP-1 and tdTomato(2xnls) levels for differences between α and β cells

We used *Fiji* (Schindelin et al. 2012) to perform image analysis. For each α and β nucleus, the somatic gonad marker expression, either mCherry::H2B (for GS9697) or mTagBFP2::H2B (for GS9911), was used to draw the segmentation boundary. The top three slices for each cell in terms of expression of their somatic gonad markers as measured by the integrated density was used to create sum *z*-projections, from which the integrated density for the somatic gonad marker, GFP::POP-1 (for both strains), and tdTomato(2xnls) (for GS9911) were measured. Background was corrected by subtracting the integrated density of an area with the same dimension as the original segmentation boundary outside the larva from the raw integrated density. Integrated density of GFP::POP-1 and tdTomato(2xnls) were normalized with mTagBFP2::H2B expression for GS9911, and integrated density of GFP::POP-1 was normalized with mCherry::H2B expression for GS9697.

### Tissue-specific depletion of LIT-1::AID in the somatic gonad

L1-arrested hermaphrodites from strains GS10126, GS10127, GS10128, and GS10129 were grown on NGM plates seeded with OP50 bacteria at 25 °C to put them past the initial division of Z1 and Z4 (11 h for GS10126, GS10127, GS10128; 12 h for GS10129) as measured by our preliminary experiments to bypass the Sys phenotype induced when Wnt signaling is abrogated in Z1 and Z4 (Miskowski et al. 2001). The larvae were then washed with M9 and pipetted into NGM OP50 plates that had been soaked with either 100 µL EtOH(100%) or 100 µL of 50 µM 5-Ph-IAA in EtOH and allowed to dry for 24–36 h. For GS10129, the larvae were grown in 25 °C for 6 additional hours (18 h total after release from L1 arrest) to analyze asymmetry of GFP::POP-1 levels between α and β nuclei in L2, while for GS10127 and GS10128, the larvae were grown for 9 additional hours in 25 °C (21 h total after release from L1 arrest) to analyze *hlh-2* transcription and HLH-2 protein levels in early L3 (T3), respectively. For GS10126, the larvae were grown for 14 additional hours in 25 °C (25 h total after release from L1 arrest), past when the somatic gonad primordium had begun to divide, to analyze specified AC fate.

The dual camera setting on the confocal microscope was used to image the somatic gonad marker *rps-27p*::mCherry(flexon) driven by *ckb-3p::*Cre(Opti) and the markers in GFP. The following exposure times were used for GS10128, GS10127, GS10129, and GS10126, respectively: mCherry: 250 ms at 10% laser power, GFP::HLH-2: 150 ms at 15% laser power; mCherry 100 ms at 10% laser power, *hlh-2p::GFP(2xnls)*: 100 ms at 15% laser power; mCherry: 100 ms at 10% laser power, GFP::POP-1: 1000 ms at 30% laser power; and mCherry: 75 ms at 10% laser power, *cdh-3::*GFP: 75 ms at 10% laser power.

*Fiji* (Schindelin et al. 2012) was used to perform image analysis. For each α and β nucleus, the somatic gonad marker mCherry was used for segmentation. The top three slices for each cell based on expression of their somatic gonad markers as measured by the integrated density was used to create sum z-projections, from which the integrated density for GFP::POP-1 (GS10129), *hlh-2p::GFP(2xnls)* (GS10127), and GFP::HLH-2 (GS10128) were also measured. Note: The AC marker *cdh-3*::GFP was assessed visually (GS10126). Background was corrected by subtracting the integrated density of an area with the same dimension as the original segmentation boundary outside the larva from the raw integrated density. For all analyses, worms with Sys phenotype gonads were excluded. The integrated density of GFP::POP-1, *hlh-2p::GFP(2xnls),* and GFP::HLH-2 were normalized to somatic gonad marker expression for each cell. For GFP::POP-1, the normalized ratio in an α nucleus/the normalized ratio of its sister β nucleus was used to assess changes in POP-1 asymmetry between the EtOH control and the 5-Ph-IAA treatment group.

### Examining *hlh-2* transcription in *lsy-12* mutants

L1-arrested larvae from strains GS10182 and GS10183 were placed onto NGM plates seeded with OP50 bacteria, grown at 25 °C, and examined under a confocal microscope after 16 and 18.5 h. GFP expression in the somatic gonad was captured at 16 h and 18.5 h after release from L1 arrest for T2 and T3, respectively, at 10% laser power and 100 ms exposure time. GFP expression was quantified using *Fiji* (Schindelin et al. 2012) using the same method as described for the deletion analysis.

### Examining effect of LIT-1::AID depletion in the *hlh-2(Δ200–250)p::gfp(2xnls)* reporter

L1-arrested GS10179 hermaphrodites were treated as described in the “Tissue-specific depletion of LIT-1::AID in the somatic gonad” section above.

## Results and discussion

### The initial expression of *hlh-2* in the α and β cells and their parents

An important goal of this study was to learn about the initial expression of *hlh-2*, which starts in Z1.pp and Z4.aa, the parents of cells that have AC potential. However, it is difficult to obtain large numbers of individuals in the T1 (parent) stage (Fig. 1b), because it is so transient, and for a given individual carrying a mutation or mutated transgene, if expression is not seen, there is also a question of whether the individual is simply too young or whether the condition truly abrogates expression. Importantly, when expression in the parents at T1 has been abrogated, expression in the daughters at stage T2 has also been abrogated, and whenever we have seen expression in T2, we have also seen it in T1. Thus, the expression in T1 and T2 appear to be linked such that together, analysis of both stages gives us information about the initial expression of genes in this paradigm.

#### Evidence for the coregulation of *hlh-2* and *nhr-67* expression in the parents of the α and β cells

*hlh-2*prox, a 327-bp element that is located 5.2 kb upstream of the start of the *hlh-2* coding region, mediates the dynamic expression pattern of *hlh-2* in the α and β cells and their parents (Sallee and Greenwald 2015; Attner et al. 2019). Mutants in which this element is disrupted in the endogenous, *gfp-*tagged gene are fully viable and fertile, but display a fully-penetrant 0 AC defect and lack visible GFP in cells of the developing proximal gonad while retaining expression in the DTCs, indicating that such “*hlh-2(Δprox)*” alleles behave as proximal gonad-specific alleles (Attner et al. 2019).

Surprisingly, given the importance of *hlh-2*prox in *hlh-2* regulation in *C. elegans*, phylogenetic analysis using *C. brenneri*, *C. briggsae*, *C. remanei*, and *C. japonica* did not identify a conserved region corresponding to *hlh-2prox.* We therefore performed an unbiased “bash” of the *hlh-2*prox element, starting with a single copy insertion transgene in a defined docking site (see Materials and Methods). This analysis identified a region of interest within *hlh-2*prox that is required to sustain *hlh-2* transcription in the α cells (considered further below) but did not identify a single subregion required for initial expression in the α and β cells and/or their parents (Supplementary Fig. 1).

We therefore considered whether another gene expressed in the α and β cells and their parents, *nhr-67*, might help illuminate elements within *hlh-2*prox. *nhr-67* encodes a nuclear hormone receptor that is homologous to human NR2E1and Drosophila Tailless (Fernandes and Sternberg 2007). Homozygotes for a deletion of *nhr-67* arrest as embryos, but the *nhr-67(pf88)* allele, a deletion of 388 nucleotides in the 5′ regulatory sequence of *nhr-67*, is viable and fertile; furthermore, the corresponding deletion mutation eliminates expression of a reporter gene (Verghese et al. 2011). Thus, *nhr-67(pf88)* has been considered to be a proximal gonad-specific strong loss-of-function or null allele (Bodofsky et al. 2018). Based on the phenotypic analysis of *nhr-67(pf88)* and other alleles associated with deletions in this region, *nhr-67* appears to contribute to the AC/VU decision but is not required for the initial potential to be an AC (Verghese et al. 2011). We note that *nhr-67* has also been studied for its roles in vulval development and later uterine patterning events (Fernandes and Sternberg 2007; Ririe et al. 2008; Verghese et al. 2011; Ranawade et al. 2013), but here, we are concerned exclusively with particular features of the earlier stages of gonadogenesis and not the later events that have already been described.

**Fig. 2.**
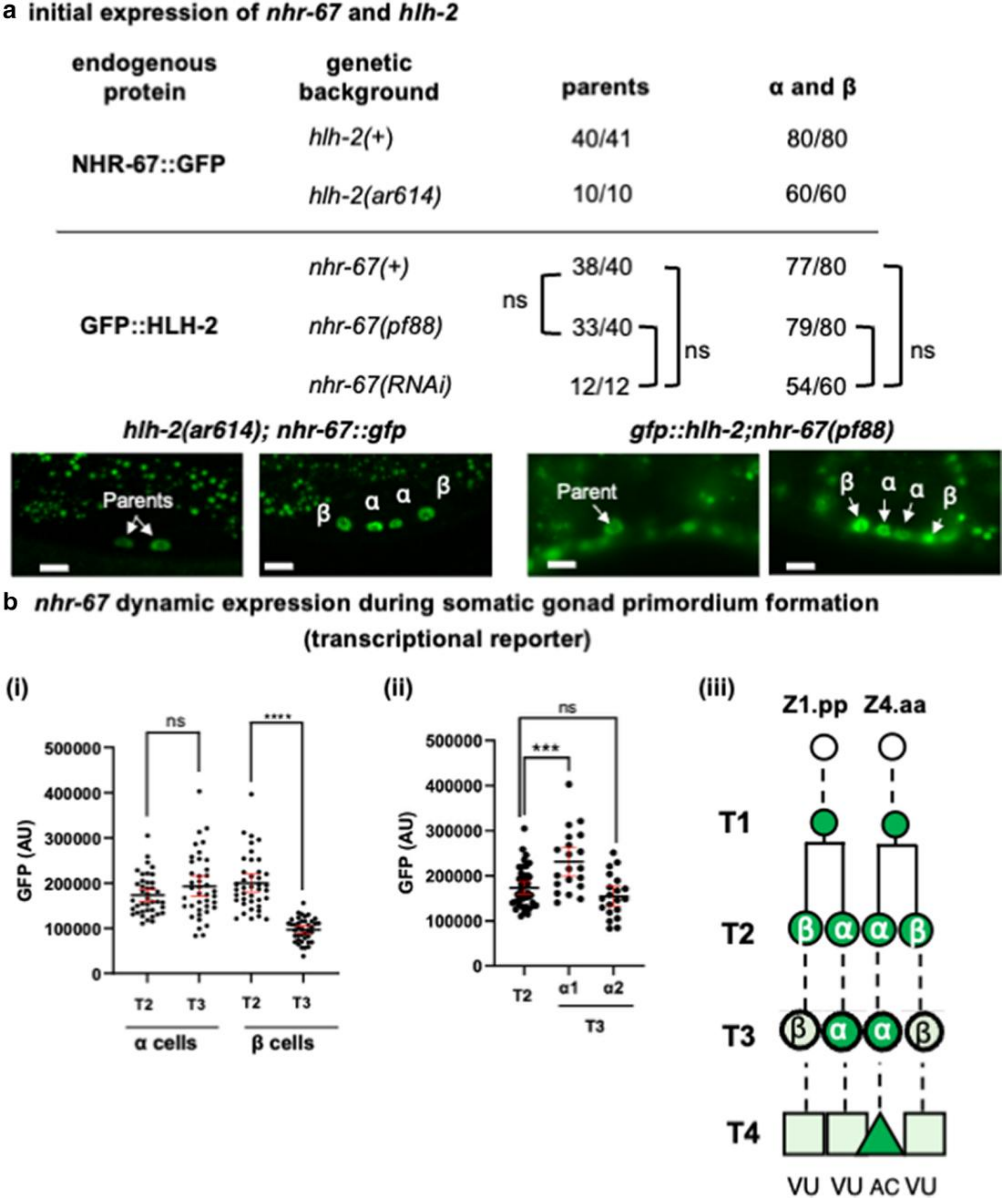
Initial evidence for the coregulation of *hlh-2* and *nhr-67* expression in the parents of the α and β cells. a) Expression of endogenous NHR-67::GFP and GFP::HLH-2. The number of cells scored is shown. If one parent had divided and the other had not, only the undivided parent was scored. Significance was assessed by the Fisher's Exact Test; ns, not significant. Representative photomicrographs are shown; here and elsewhere, scale bar indicates 5 µm unless otherwise indicated. b) *nhr-67* dynamic expression during somatic gonad primordium formation. (i) GFP expression of the transcriptional reporter *arSi194[nhr-67p::gfp(2xnls)]*. Each point represents a cell. (ii) Data for α cells from (i) at the T3 timepoint, showing the increase in one of the two α cells over time from T2. In each animal, the higher-expressing a cell was designated α1, and the lower-expressing a cell was designated α2. (iii) Time course of *arSi194[nhr-67p::gfp(2xnls)]* expression. The transcriptional reporter is expressed in the parents at T1, the alpha and beta cells at T2, and the alpha cells at T3. At T4, GFP expression in the αVU is inconsistent: in 14/20 hermaphrodites, only one cell expressed bright GFP and in 6/20 hermaphrodites, both α cells still expressed GFP. Statistical tests for (i) and (ii) were Mann–Whitney *U* tests. ***P < 0.001, ****P < 0.0001; ns, not significant.

**Fig. 3.**
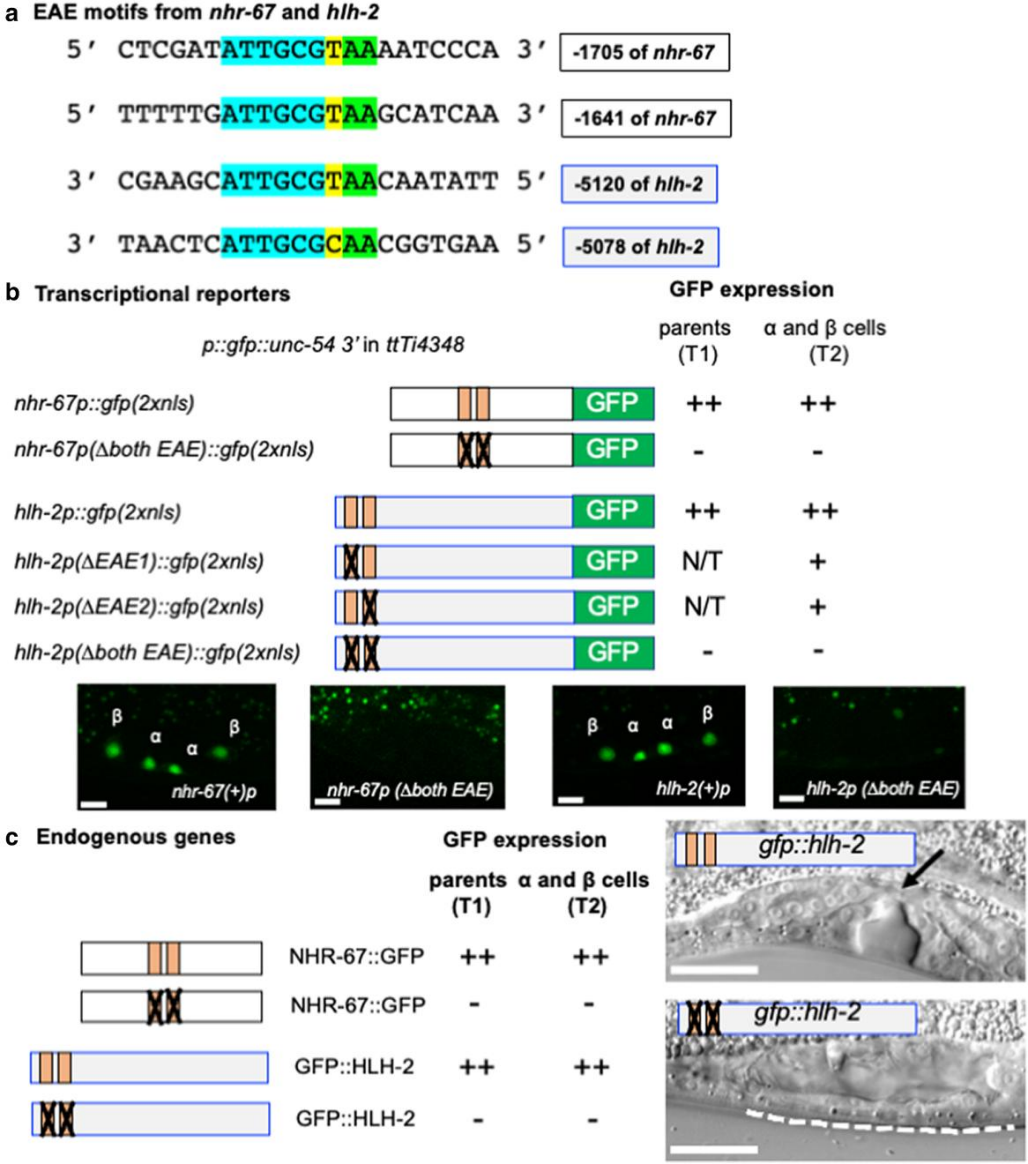
EAE elements are required for initial *hlh-2* and *nhr-67* transcription. a) Alignment of the EAE sites. The EAE element defined by Bodofsky et al. (2018), ATTGCGY is shared between the *hlh-2*prox and *nhr-67* 5′ upstream regions (identities are highlighted in blue, the Y is highlighted in yellow). Here we have extended the sequence of the EAE by conserved “AA” (highlighted in green). The adjacent number indicates the position upstream of the respective ATG. b) Mutation of EAE sites abrogates initial transcription of *nhr-67* and *hlh-2*. The wild-type *nhr-67p::gfp(2xnls) (arSi194)* reporter is expressed in α and β cells at T2 (76/76 cells) and their parents at T1 (32/33 cells). In the mutant *nhr-67(Δboth EAE)p::GFP(2xnls)* reporter (*arSi196)*, GFP expression absent from α and β cells at T2 (75/80 cells) and their parents at T1 (42/42 cells). ++ indicates visibly bright expression. The wild-type *hlh-2p::gfp(2xnls)* (*arSi155*) reporter is expressed in α and β cells at T2 (80/80 cells) and in their parents (21/21). ++ indicates visibly bright expression. Reporters with single EAE sites mutated, *hlh-2p(ΔEAE1)::gfp(2xnls)* (*arSi183)* and *hlh-2p(ΔEAE2)::gfp(2xnls)* (*arSi175)* displayed detectable but significantly lower level of transcription, represented by + instead of ++ (Supplementary Fig. 2). When both EAEs are mutated, *hlh-2p(Δboth EAE)::gfp(2xnls)* (*arSi174)*, GFP expression was not visible in 84/88 of α and β cells at T2 and not visible at all at T1 (44/44). N/T indicates not tested. c) Mutation of EAE sites abrogates endogenous NHR-67::GFP and GFP::HLH-2 expression. Endogenous NHR-67::GFP is expressed in α and β cells and their parents in *nhr-67(syb509)* (see Fig. 2). When both EAEs are mutant [*nhr-67(syb509ar667)*], NHR-67::GFP is not expressed in the parents of the α and β cells (0/47 cells) or in the α and β cells (78/80, no expression). ++ indicates bright expression. Endogenous GFP::HLH-2 [*hlh-2(ar623)*] is expressed in α and β cells and their parents in *gfp::hlh-2 [hlh-2(ar623)]* (see Fig. 2). When both EAEs are mutant *[hlh-2(ar623ar668)]* GFP::HLH-2 is not expressed in the parents of the α and β cells (0/63 cells) or α and β cells (74/80 no expression). ++ indicates bright expression. In L4 hermaphrodites (photomicrographs), a vulval invagination is a proxy for AC function; an invagination was observed in 20/20 of *hlh-2(ar623)* hermaphrodites whereas 15/20 of *hlh-2(ar623ar668)* hermaphrodites, with both EAEs mutant, lacked an invagination (the other 5/20 were wild-type). Scale bar indicates 20 µm.

Dissection of the cis-regulatory region of *nhr-67* using multicopy transcriptional reporters had identified potential regulatory elements within the region defined by *nhr-67(pf88)* (Bodofsky et al. 2018). We found that these elements are also present in *hlh-2*prox (characterized below and shown in Fig. 3a), suggesting potential coregulation of *hlh-2* and *nhr-67* via these elements in the α and β cells or their parents. If so, then *hlh-2* and *nhr-67* should not be required for the expression of each other during the formation of the somatic gonad primordium, even though in the L3 stage, *hlh-2* promotes *nhr-67* expression in the AC (Medwig-Kinney et al. 2023). Indeed, RNAi experiments suggested such independence at an early stage (Medwig-Kinney et al. 2023), but given the caveats in interpreting negative RNAi results, we wanted to corroborate this inference using the proximal gonad-specific loss-of-function alleles, scoring expression in α and β cells and their parents without regard to cell fate (Fig. 2a). Loss of *hlh-2* activity in the *hlh-2(Δprox)* allele *hlh-2(ar614)* (Attner et al. 2019) did not prevent the expression of endogenous NHR-67::GFP in the α and β cells and their parents through somatic gonad primordium formation. Similarly, loss of *nhr-67* activity in *nhr-67(pf88)* (Verghese et al. 2011) did not prevent endogenous GFP::HLH-2 expression in the α and β cells and their parents through somatic gonad primordium formation.

We also looked more closely *nhr-67* transcription and endogenous NHR-67::GFP expression, verifying that both are initially expressed in the parents and in the α and β cells (Fig. 3b and 3c). The *nhr-67* transcriptional reporter (Fig. 2b) and endogenous NHR-67::GFP (Medwig-Kinney et al. 2023) become enriched in the AC compared with the VU by the time the somatic primordium has formed, in contrast to the *hlh-2* transcriptional reporter, which continues to be expressed in both α cells while GFP::HLH-2 becomes restricted to the AC by being degraded in VUs (Fig. 1c).

Together, the mutant analysis and expression time course provide strong evidence that *hlh-2* and *nhr-67* do not regulate each other's initial expression, consistent with the possibility that they are instead co-regulated.

#### Two Early Activation Elements are required for the initial expression of endogenous *nhr-67* and *hlh-2* in the α and β cells and their parents

Loss of *nhr-67* function is lethal, but *nhr-67(pf88)* is viable and displays the aforementioned 2 AC defect. Using phylogenetic analysis comparing *C. elegans* with *C. brenneri*, *C. briggsae*, and *C. remanei*, Bodofsky et al. (2018) identified two copies of the element “ATTGCGY” (Y = C or T) within the region deleted by *nhr-67(pf88)* and showed that these elements are necessary for the expression of *nhr-67p::gfp* multicopy transcriptional reporters in α and β cells. We found that there are also two copies of the “ATTGCGY” sequence within *hlh-2*prox and note that in both *nhr-67* and *hlh-2*, the full sequence element appears to be “ATTGCGYAA” (Fig. 3a).

Using genetic engineering tools that have become available since the work of Bodofsky et al. (2018), we tested whether the two ATTGCGY sites in *nhr-67* and *hlh-2* act as Early Activation Element (EAEs), i.e. required for the initial expression of these genes. In the context of single-copy insertion transcriptional reporters in the defined docking site, we found that mutating both EAE sites abrogated expression in the parents and initially in the α and β cells, suggesting they act as EAEs (Fig. 3b), while mutating individual EAE sites in *hlh-2* partially reduced expression (Supplementary Fig. 2). When both EAEs were mutated in endogenous *nhr-67::gfp* [*nhr-67(syb509ar667)*], NHR-67::GFP expression was not detected in the parents of the α and β cells and very rarely seen in the α and β cells (Fig. 3c). Similarly, when both EAEs were mutated in endogenous *gfp::hlh-2* [*hlh-2(ar623ar668)*], GFP::HLH-2 expression was not detected in the parents of the α and β cells and only rarely observed in the α or β cells (Fig. 3c). Thus, our analysis indicates that the ATTGCGYAA sequences act as EAEs in both *nhr-67* and *hlh-2*.

We note that when both EAEs were mutated in endogenous *hlh-2*, 75% (15/20) of the hermaphrodites were Vulvaless, consistent with the lack of an AC as would be expected if early *hlh-2* expression is lost (Fig. 3c). However, a deletion removing most of *hlh-2*prox causes a completely penetrant Vulvaless phenotype (Attner et al. 2019), suggesting that other elements in *hlh-2*prox may contribute to early *hlh-2* expression or that the mutations did not fully compromise these elements.

We also note the possibility that mutating both *nhr-67* EAEs, thereby reducing its initial expression, appeared to prolong the time it took for α and β cells to resolve their fates. This possibility is based on our conclusion that the initial expression of *nhr-67* does not depend on *hlh-2* activity. The pattern of HLH-2 protein accumulation can serve as a marker for the progression of the AC/VU decision: initially present in the four α and β cells, then restricted to just the α cells, and finally, just to the AC. Thus, in T4 stage hermaphrodites, in an *nhr-67(+)::gfp* background, endogenously-tagged wrmScarlet::HLH-2 was evident only in the presumed AC (19/20). By contrast, in T4 stage mutant hermaphrodites lacking the *nhr-67* EAEs, wrmScarlet::HLH-2 expression appeared to persist in a majority of hermaphrodites: in the four α and β cells (4/20), three cells (8/20), and two cells (6/20), with only 2/20 animals showing the normal restriction to just the AC. This result is consistent with irresolution of the AC/VU decision when early expression of *nhr-67* is compromised, in accordance with a proposed role of *nhr-67* in promoting the VU fate during the AC/VU decision (Verghese et al. 2011).

#### RNAi screen for transcription factors that may act via the EAEs of *hlh-2* and *nhr-67*

**Fig. 4.**
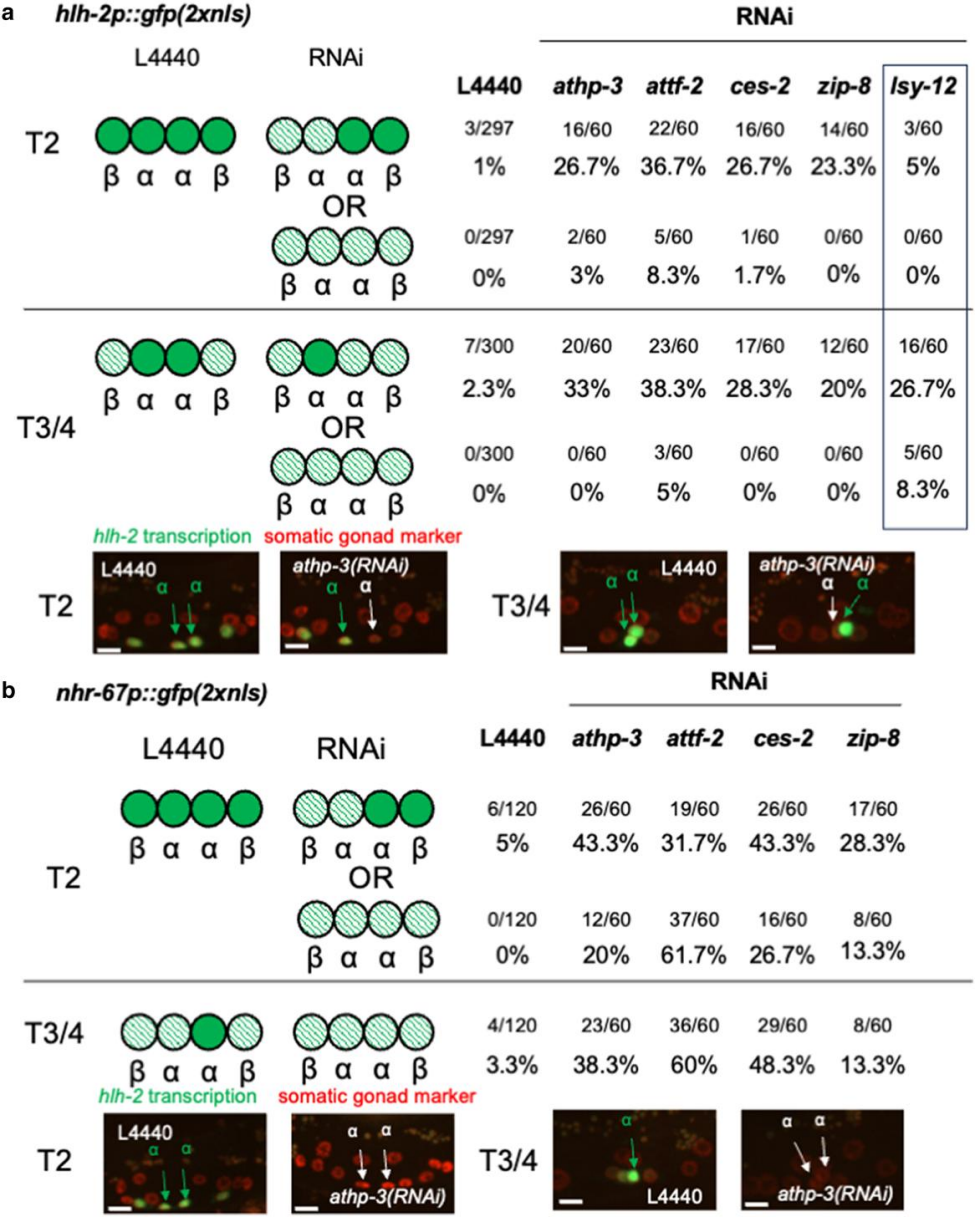
RNAi screen of candidate AT-hook and bZIP transcription factors. Representative images are shown for *athp-3(RNAi)* and the negative control, L4440 empty-vector bacteria. Data for the *lsy-12* gene (boxed) are included here for convenience but will be discussed later. a) Screen for effects on *hlh-2p::GFP(2xnls)*. GS10123 larvae were treated with feeding RNAi and scored by eye at T2 and T3/4. The negative control is bacteria containing the empty vector, L4440. The cartoons indicate the phenotype, with full green indicating bright expression. The fraction indicates the number of animals with the indicated phenotype/total scored. Each RNAi condition was tested in triplicate, in batches of 20, and the pooled numbers are shown, with the corresponding percentages below, b) Test of positive genes from A for effects on *nhr-67p::GFP(2xnls*). GS10160 larvae were tested in triplicate.

The “ATTGCGYAA” sequence of the EAEs resembles the consensus DNA binding site of mammalian AT-hook protein HMGA2 (TATTGCGCAWWATT) (Cui and Leng 2007) (W = A/T), and the consensus DNA binding site of the C/EBP family of bZIP transcription factors “ATTGCGCAAT” (Vinson et al. 1989). The transcription factor binding prediction tool CIS-BP (Weirauch et al. 2014) found the top five candidates for regulators that act via the EAEs to be *athp-3* and *hmg-12*, which encode AT-hook transcription factors, and *atf-2*, *ces-2*, and *zip-8*, which encode bZIP transcription factors. There are also 26 predicted AT-hook transcription factors and the 4 C/EBP bZIP orthologs not present in CIS-BP. RNAi depletion targeting AT-hook and bZIP genes in a strain carrying the wild-type *hlh-2* transcriptional reporter and the RNAi-sensitizer *nre-1(hd20) lin-15B(hd126)* (Schmitz et al. 2007) revealed that depletion of *athp-3*, *ces-2, and zip-8* predicted by CIS-BP and an additional AT-hook protein-encoding gene, *attf-2*, reduced early *hlh-2* transcription (Fig. 4a).

In view of our working hypothesis that initial *hlh-2* and *nhr-67* transcription is coregulated, we then tested the effect of *athp-3(RNAi)*, *attf-2(RNAi)*, *ces-2(RNAi),* and *zip-8(RNAi)* on expression of the wild-type *nhr-67* transcriptional reporter in the RNAi-sensitized background. RNAi targeting all four genes resulted in reduced *nhr-67* transcription compared with the empty vector negative control (Fig. 4b). Indeed, *nhr-67* transcription appears to be more strongly affected than *hlh-2* transcription, but the mechanistic basis is not clear; for example, while the EAEs are the same, the context in which they occur is different, so the contribution of any given factor may be different in its context.

The finding that all candidates identified by reducing *hlh-2* expression also reduce *nhr-67* expression supports the hypothesis that initial expression of *hlh-2* and *nhr-67* are co-regulated. It also suggests that one or more of these genes are a good candidate for mediating activation through the EAEs, although we cannot conclude based on these data alone that any or all of these genes are direct activators of *hlh-2* and *nhr-67* via the EAEs.

We also note that despite depletion of these transcription factors, in the L3 stage, RNAi-treated animals had a single GFP::HLH-2-expressing AC (data not shown), suggesting that there was sufficient residual activity either due to incomplete depletion or genetic redundancy to confer AC potential and to resolve the AC/VU decision. Similarly, incomplete depletion or genetic redundancy may account for the incompletely penetrant phenotypes.

### The α/β difference in *hlh-2* transcription is regulated by the Wnt/β-catenin asymmetry pathway

The Wnt/β-catenin Asymmetry Pathway (WβA) in *C. elegans* creates differences between sister cells in many lineages: asymmetric localization of proteins in a parent cell results in differing levels of Wnt pathway genes in the daughter cells, including the level of the transcription factor POP-1/TCF in the daughter cells [reviewed in (Sawa and Korswagen 2013)]. In WβA, the kinase LIT-1, the ortholog of mammalian NLK, phosphorylates nuclear POP-1 to promote its export to the cytoplasm, thus lowering the relative level of nuclear POP-1 (Rocheleau et al. 1999; Lo et al. 2004). Here, we test the model suggested by previous studies that the α/β difference in *hlh-2* transcription is caused by a difference in the level of POP-1 (Sallee et al. 2015; Benavidez et al. 2022).

#### The asymmetrically lower level of GFP::POP-1 in β cells precedes loss of *hlh-2* transcription

**Fig. 5.**
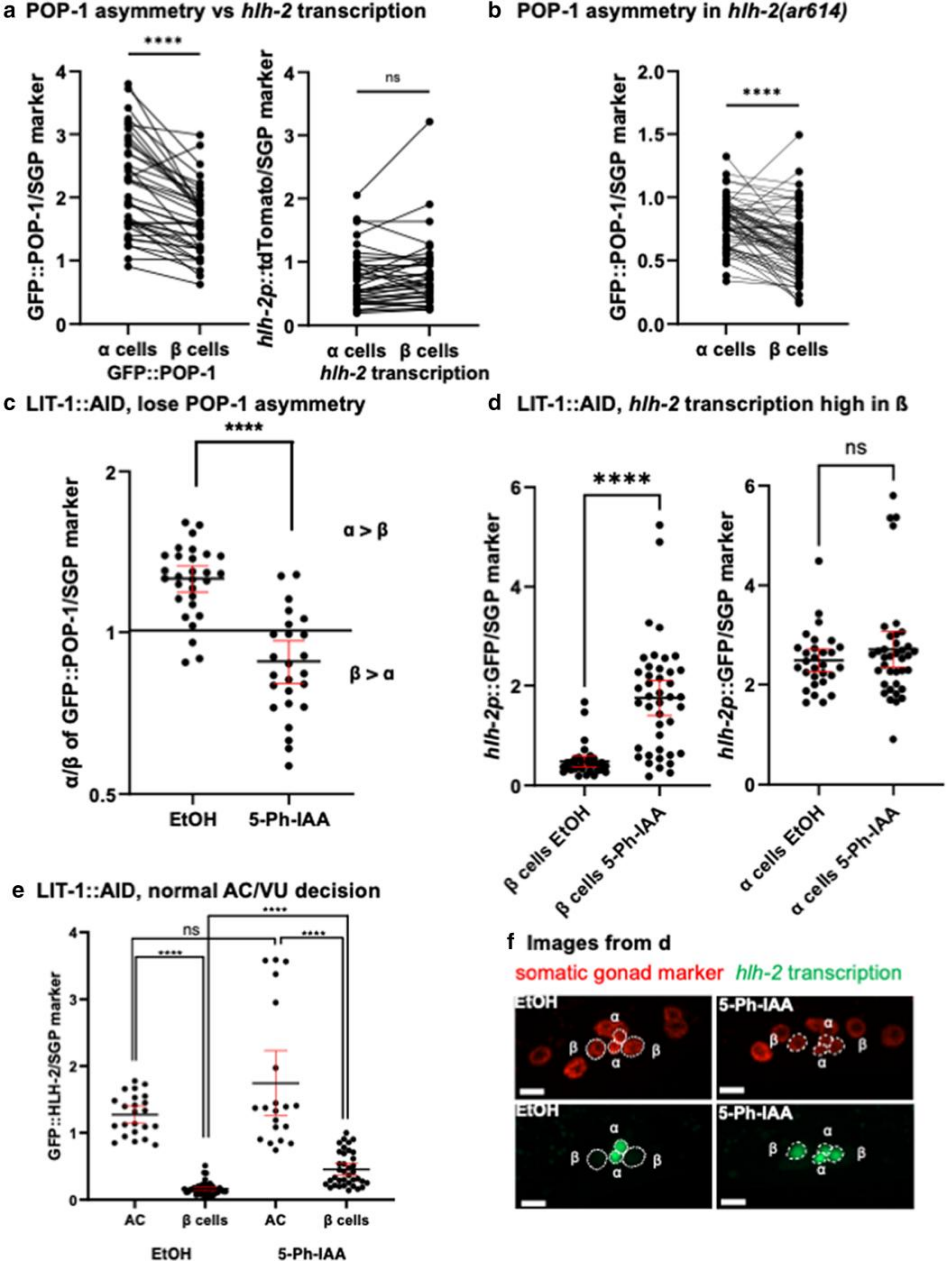
The α/β difference in *hlh-2* transcription is regulated by the WβA pathway. a) GFP::POP-1 accumulates at higher levels in α cell nuclei compared with β cell nuclei. Left: Endogenous GFP::POP-1 level normalized to a somatic gonad marker *arTi448* (see Methods) is higher in α cell nuclei than in β cell nuclei. Right: In the same cells analyzed for GFP::POP-1 tdTomato expression from *arT1460 [hlh-2p::tdTomato(2xnls)]* was normalized to *arTi448*, showing that transcription of *hlh-2* is similar in α and β cell. *n = 21* animals. Thus, the asymmetry in GFP::POP-1 levels precedes loss of *hlh-2* transcription in β cells. Statistical test, Wilcoxon matched-pairs signed rank tests. ****P < 0.0001; ns, not significant. b) GFP::POP-1 asymmetry does not depend on *hlh-2* activity. In the *hlh-2(ar614)* background, endogenous GFP::POP-1 level normalized to somatic gonad marker levels is generally higher in α cell nuclei than in β cell nuclei. *n = 32* animals. Statistical test, Wilcoxon matched-pairs signed rank tests. ****P < 0.0001. c) Loss of LIT-1 results in loss of POP-1 asymmetry. The level of GFP::POP-1 in α and β cells was first normalized to the somatic gonad marker and then the normalized α/β ratio was calculated. EtOH is the negative control for the effect of 50 µM 5-Ph-IAA treatment. *n = 15* animals for EtOH, *n = 13* animals for 5-Ph-IAA. Statistical test, Mann–Whitney *U* tests. ****P < 0.0001. Black bars indicate mean value, and red bars indicate 95% confidence intervals. d) Loss of POP-1 asymmetry stabilizes *hlh-2* transcription in β cells. Left, normalized transcription of *arSi155* [*hlh-2p::gfp(2xnls)]* in the absence (EtOH) or presence (5-Ph-IAA) of auxin at T4 in β cells. Right, normalized transcription of *arSi155* [*hlh-2p::gfp(2xnls)]* in the absence (EtOH) or presence (5-Ph-IAA) of auxin at T4 in α cells. *n = 17* for EtOH, *n = 21* for 5-Ph-IAA. Statistical test, Mann–Whitney *U* tests. ****P < 0.0001; ns, not significant. Black bars indicate mean value, and red bars indicate 95% confidence intervals. e) Stabilization of POP-1 does not abrogate the AC/VU decision. At T4, a visible difference in GFP::HLH-2 expression in a single cell was inferred to be evidence that the AC/VU decision had occurred normally. The α cell expressing the highest level of GFP::HLH-2 was designated the AC. Pairwise comparisons suggest that no β cells achieved a comparable level of GFP::HLH-2 expression, suggesting they were not transformed into ACs. This inference was supported by an independent marker, *arIs51[cdh-3::gfp]*, which was strongly expressed in only one cell (20/20, strain GS10126). Statistical test for comparison across treatment groups, Mann–Whitney *U* tests. ****P < 0.0001; ns, not significant. Black bars indicate mean value, and red bars indicate 95% confidence intervals. Statistical test for comparison within a treatment group, Wilcoxon matched-pairs signed rank tests. ****P < 0.0001; ns, not significant. f) Representative images from D.

If the loss of *hlh-2* transcription in the β cells is a consequence of the lower level of POP-1 in these cells compared with α cells, we would expect that the asymmetric distribution of endogenous GFP::POP-1 would precede the loss of *hlh-2* transcription in the β cells and would not depend on *hlh-2* activity. These expectations were met: the level of endogenous GFP::POP-1 is lower in β cells when *hlh-2p::tdTomato(2xnls)* encoded by an *hlh-2* transcriptional reporter is at similar levels between α and β cells (Fig. 5a) and endogenous GFP::POP-1 asymmetry is still observed in *hlh-2(ar614)*, the proximal gonad-specific null allele of *hlh-2* (Fig. 5b).

#### Depleting LIT-1::AID stabilizes GFP::POP-1 and sustains *hlh-2* transcription in β cells

If POP-1 asymmetry governs the differential transcriptional regulation of *hlh-2* in α and β cells, increasing the level of POP-1 in β cells should sustain *hlh-2* transcription. To stabilize GFP::POP-1, we tagged endogenous LIT-1 with the Auxin Inducible Degron (AID) and achieved robust somatic gonad-specific expression of TIR1F79G using a Flexon-based system (Materials and Methods), adding auxin after the first division of Z1 and Z4 to bypass a requirement for WβA that would otherwise mask a later role in the lineage. We found that LIT-1::AID depletion abrogates GFP::POP-1 nuclear asymmetry between the α and β cells (Fig. 5c) and that GFP(2xNLS) produced from the *hlh-2* transcriptional reporter persisted in the β cells during stage T4, when it would normally never be seen (Fig. 5d and f). Thus, we conclude that WβA normally leads to the loss of POP-1 in β cells and to loss of *hlh-2* expression. The mechanism underlying loss of *hlh-2* expression is likely to be indirect, because *hlh-2*prox lacks the binding consensus sequence for TCF/LEF1, which binds a POP-1 complex with a β-catenin, as in the relevant target *ceh-22* in Z1 and Z4 (Lam et al. 2006) and also lacks a Zic binding site, which binds a POP-1 complex with REF-2 (Bertrand and Hobert 2009).

Depletion of LIT-1::AID also resulted in persistent expression of endogenously-tagged GFP::HLH-2 expression in βVUs compared with the control (Fig. 5e). However, GFP::HLH-2 expression in βVUs was nevertheless lower than that in the AC, suggesting a lack of βAC transformation. Thus, while HLH-2 is necessary to endow AC potential, it is not sufficient to cause AC fate, consistent with the lack of cell fate transformation observed when HLH-2 protein is stabilized by depleting ubiquitin ligases (Benavidez et al. 2022).

### The maintenance of *hlh-2* transcription in α cells requires elements that are distinct from the EAEs

#### Identification of α cell maintenance elements

**Fig. 6.**
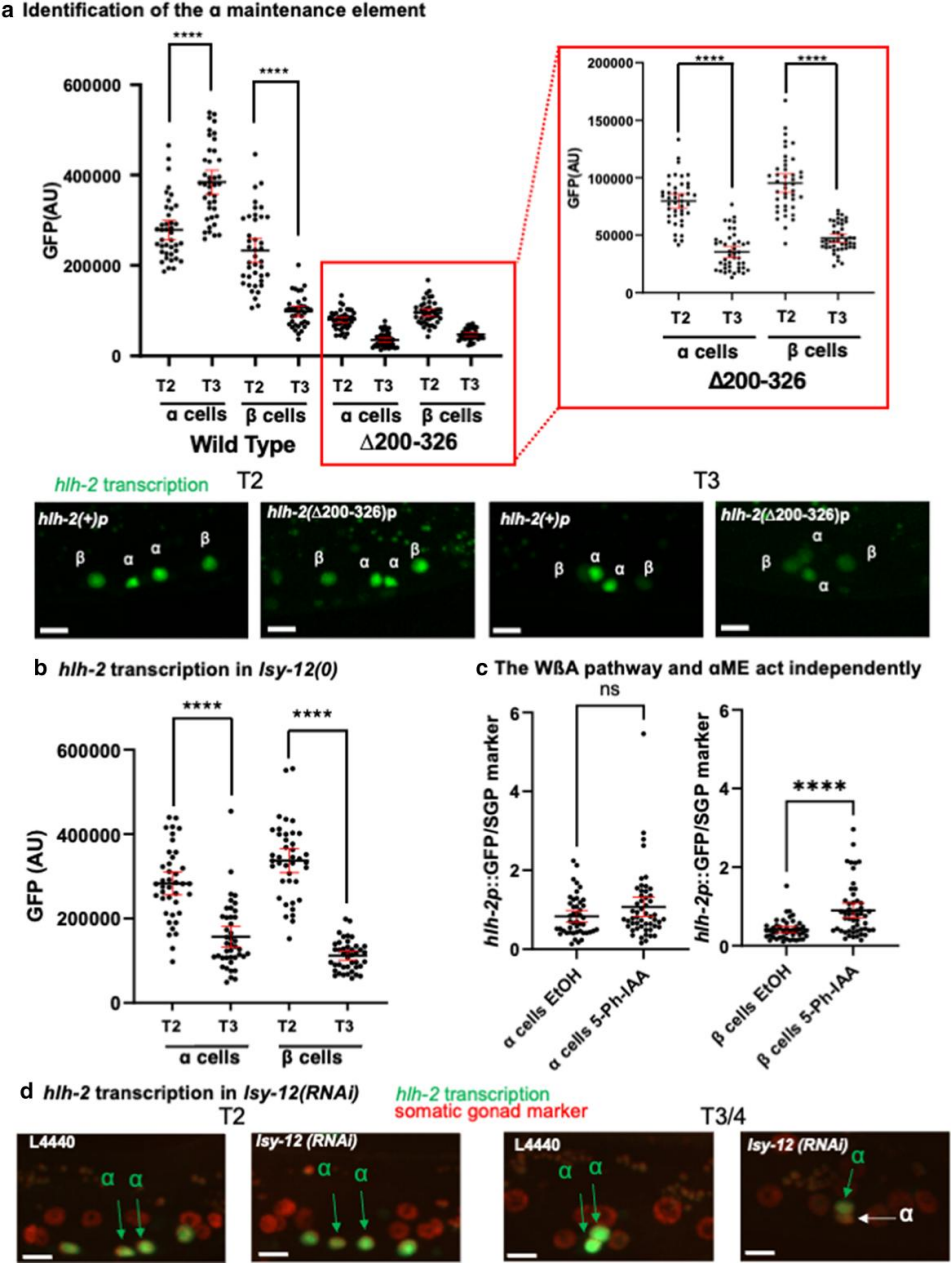
Maintenance of *hlh-2* transcription in α cells. a) Identification of the α Maintenance Element (αME). Deletion of nucleotides 200–326 of *hlh-2*prox in a transcription reporter lowers transcription overall, but the loss of *hlh-2* transcription in α cells in T3 compared with T2 is evident, suggesting that this region is contributing to sustained transcription of *hlh-2* in α cells. (Wild-type, *n = 20* for T2, *n = 20* for T3; Δ200–326, (*n = 21* for T2, *n = 22* for T3). Further deletion analysis within this region identified two subregions that each behaved like the 200–326 deletion (see Supplementary Fig. 1). Right, expanded view of Δ200–326 quantification Black bars indicates mean value, and red bars indicate 95% confidence intervals. Statistical test, Mann–Whitney *U* tests. ****P < 0.0001. b) *lsy-12* is required for maintenance of *hlh-2* transcription in α cells. *hlh-2* transcription normally increases from T2 to T3 in α cells (see Fig. 6a). However, in the *lsy-12(ot170)* null mutant background, *hlh-2* transcription decreases in α cells from T2 to T3, indicating that it is not maintained. Similar results were obtained for another null allele, *lsy-12(ot171)* (data not shown). Black bars indicate mean value, and red bars indicate 95% confidence intervals. Statistical test, Mann–Whitney *U* tests. **P < 0.01, ***P < 0.001, ****P < 0.0001. c) Evidence that the WßA pathway and ɑME act independently. Left, expression from the *arSi169* Δ200–250 *hlh-2* transcriptional reporter, which lacks element(s) needed for sustained *hlh-2* expression in α cells, was normalized to the somatic gonad marker expression. Right, expression increases in β cells when LIT-1::AID is concomitantly depleted. *n = 23* for EtOH, *n = 26* for 5-Ph-IAA. Black bars indicate mean value, and red bars indicate 95% confidence intervals. Statistical tests for a and b were Mann–Whitney *U* tests. **P < 0.01, ***P < 0.001, ****P < 0.0001. d) Representative images showing the effect of reduced *lsy-12* activity. L4440 is the negative control empty-vector bacterial strain.

*hlh-2* transcriptional reporter expression not only decreases in the β cells but also increases in the α cells over time as the somatic gonad primordium forms, which likely correlates with the engagement of the LIN-12/Notch-mediated AC/VU decision in the α cells (Fig. 6a). In the initial “bash” of *hlh-2*prox, the Δ200–326 reporter led to reduced instead of increased expression in the α cells compared with the β cells (Fig. 6a). Two smaller deletions, Δ200–250 and Δ286–326, showed a similar pattern (Supplementary Fig. 3), suggesting that they contain elements needed to maintain *hlh-2* transcription in α cells.

#### *lsy-12* is required for maintenance of *hlh-2* transcription in α cells

While screening predicted AT-hook transcription factors by RNAi for loss of the initial transcription of *hlh-2*, we also examined older animals for an effect on expression at a time when *hlh-2* is normally transcribed only in the α cells. *lsy-12(RNAi)* stood out in that it appeared to diminish *hlh-2* transcription only at this time, not earlier (Fig. 4a). We confirmed this observation using two inferred loss-of-function mutations, *lsy-12(ot170)* and *lsy-12(ot171)* (Sarin et al. 2007): the *hlh-2* transcriptional reporter is initially robustly expressed in the four α and β cells and decreases not just in β cells (presumably due to WβA as described above) but also in the α cells (Fig. 6b), as in the ΔαME transcriptional reporters.

We also examined expression of the Δ200–250 *hlh-2* transcriptional reporter, which lacks the element(s) needed for sustained *hlh-2* expression in α cells, while also depleting LIT-1::AID. Transcription in α cells was not affected, but the level in β cells was increased, suggesting that the WßA pathway and ɑME act independently (Fig. 6c).

### Concluding remarks: rapid evolution of *hlh-2* regulation in gonadogenesis

The AC is a critical signaling nexus for vulval and uterine fate patterning in *C. elegans*, and *hlh-2* is critical for AC specification and function. However, although the *hlh-2*prox element of *C. elegans hlh-2* orchestrates the dynamic pattern of *hlh-2* transcription in the life history of the AC, we were unable to identify *hlh-2*prox-like regions in the *hlh-2* orthologs of several *Caenorhabditis* species. At the outset of this study, this lack of conservation necessitated a different approach to identifying features of the cis-regulatory architecture of *C. elegans hlh-2*, but we want to conclude here by considering the evolutionary implications for *hlh-2* regulation.

Although we do not have experimental evidence that *hlh-2* plays the same role in AC specification in other *Caenorhabditids* as in *C. elegans*, a comprehensive study by (Barkoulas et al. 2016) of the regulation of the HLH-2 transcriptional target *lin-3* suggests that at least this important role is conserved. *lin-3* encodes the EGF-like ligand that mediates vulval induction (Hill and Sternberg 1992) and in *C. elegans*, the critical regulatory element necessary for *lin-3* expression in the AC consists of two E-boxes that flank a binding site for the nuclear hormone receptor NHR-25 (Hwang and Sternberg 2004). By contrast, Barkoulas et al. (2016) have shown that, while *lin-3* is expressed at similar levels in the AC of different *Caenorhabditis* species, in *Caenorhabditis angaria* only a single E-box is needed, suggesting there is considerable plasticity in the *cis*-regulatory information that can be used for *lin-3* regulation by HLH-2. They explain this plasticity by proposing that compensatory evolution has occurred, obviating the need for a second E-box and the NHR binding site in *lin-3* of *C. angaria*. Perhaps such compensatory evolution has also occurred in the regulatory sequences that govern *hlh-2* expression during proximal gonadogenesis.

## Supplementary Material

jkaf288_Supplementary_Data

## Data Availability

Strains and plasmids are available upon request. The authors affirm that all data necessary for confirming the conclusions of the article are present within the article, figures, and tables. Supplemental material available at G3 online.
