# Supplementary material for: Regulation of hlh-2 transcription during specification of the anchor cell of the C. elegans hermaphrodite gonad
Source: G3 (Bethesda). 2025 Dec 2;16(2):jkaf288. doi: 10.1093/g3journal/jkaf288 (PMC12869078; doi:10.1093/g3journal/jkaf288)
Supplement: jkaf288_Supplementary_Data [file jkaf288_supplementary_data.zip › Supplemental_Tables_G3-2025-406240.docx]

**Supplemental Table 1. Alleles and transgenes.** * *arSi214* was derived from the same unexcised SEC strain as *arSi194.*

| **Allele** | **Genotype** | **Reference** |
| --- | --- | --- |
| *ar614* | *hlh-2(ar614) I* | Attner et al. 2019 |
| *ar623* | *hlh-2{ar623[gfp::hlh-2]} I* | Attner et al. 2019 |
| *ar657* | *hlh-2{ar657[wrmScarlet::hlh-2]} I* | This paper |
| *ar665* | *lit-1{ar665[lit-1::AID]} III* | This paper |
| *syb509ar667* | *nhr-67{syb509ar667[nhr-67p(∆both EAE)::nhr-67::gfp]} IV* | This paper |
| *ar623ar668* | *hlh-2{ar623ar668[hlh-2p(∆both EAE)::gfp::hlh-2]} I* | This paper |
| *arIs51* | *cdh-3::gfp IV* | Karp and Greenwald 2003 |
| *arSi138* | *hlh-2p(5.2kb hlh-2prox ∆1-100)::gfp(2xnls)::unc-54 3'UTR I* | This paper |
| *arSi144* | *hlh-2p(5.2kb hlh-2prox ∆150-250)::gfp(2xnls)::unc-54 3'UTR I* | This paper |
| *arSi145* | *hlh-2p(5.2kb hlh-2prox ∆50-150)::gfp(2xnls)::unc-54 3'UTR I* | This paper |
| *arSi154* | *hlh-2p(5.2kb hlh-2prox ∆200-326)::gfp(2xnls)::unc-54 3'UTR I* | This paper |
| *arSi155* | *hlh-2p(5.2kb)::gfp(2xnls)::unc-54 3' UTR I* | This paper |
| *arSi156* | *hlh-2p(5.2kb hlh-2prox ∆100-200)::gfp(2xnls)::unc-54 3'UTR I* | This paper |
| *arSi164* | *hlh-2p(5.2kb hlh-2prox ∆286-326)::gfp(2xnls)::unc-54 3'UTR I* | This paper |
| *arSi169* | *hlh-2p(5.2kb hlh-2prox ∆200-250)::gfp(2xnls)::unc-54 3'UTR I* | This paper |
| *arSi174* | *hlh-2p(5.2kb hlh-2prox ∆both EAE)::gfp(2xnls)::unc-54 3'UTR I* | This paper |
| *arSi175* | *hlh-2p(5.2kb hlh-2prox ∆EAE2)::gfp(2xnls)::unc-54 3'UTR I* | This paper |
| *arSi183* | *hlh-2p(5.2kb hlh-2prox ∆EAE1)::gfp(2xnls)::unc-54 3'UTR I* | This paper |
| *arSi194* | *nhr-67p::gfp(2xnls)::unc-54 3'UTR I* | This paper |
| *arSi196* | *nhr-67p(∆both EAE)::gfp(2xnls)::unc-54 3'UTR I* | This paper |
| *arSi214* | *nhr-67p::gfp(2xnls)::unc-54 3'UTR I ** | This paper |
| *arTi112* | *ckb-3p::mCherry::his-58::unc-54 3’UTR V* | Attner et al. 2019 |
| *arTi145* | *ckb-3p::mCherry::his-58::unc-54 3’UTR II* | Attner et al. 2019 |
| *arTi237* | *ckb-3p::Cre(opti)::tbb-2 3’UTR X* | Shaffer and Greenwald 2022a |
| *arTi443* | *rps-27p::TIR1F79G9(flexon)::unc-54 3’UTR V* | Wittes and Greenwald 2024 |
| *arTi448* | *ckb-3p::mTagBFP2::his-11::unc-54 3’UTR II* | This paper |
| *arTi460* | *hlh-2p(5.2kb)::tdTomato(2xnls)::unc-54 3’UTR III* | This paper |
| *arTi481* | *rps-27p::mCherry(flexon)::his-58::unc-54 3’UTR I* | This paper |
| *arTi483* | *rps-27p::mCherry(flexon)::his-58::unc-54 3’UTR IV* | This paper |
| *he335* | *pop-1{he335[egfp::pop-1]} I* | van der Horst et al. 2019 |
| *ot170* | *lsy-12(ot170) V* | Sarin et al. 2007 |
| *ot171* | *lsy-12(ot171) V* | Sarin et al. 2007 |
| *pf88* | *nhr-67(pf88) IV* | Verghese et al. 2011 |
| *syb509* | *nhr-67{syb509[nhr-67::gfp]} IV* | Medwig-Kinney et al. 2020 |

**Supplemental Table 2. Strains.** GS8995 was described in Benavidez et al. (2022). All other strains were generated for this study.

| **Strain** | **Genotype** |
| --- | --- |
| GS8995 | *hlh-2(ar623);arTi145;nre-1(hd20)lin-15B(hd126)* |
| GS9222 | *hlh-2(ar623);arTi112* |
| GS9697 | *hlh-2(ar614) pop-1(he335);arTi145* |
| GS9911 | *pop-1(he335);arTi448;arTi460* |
| GS10001 | *arSi138* |
| GS10020 | *arSi144* |
| GS10021 | *arSi145* |
| GS10031 | *arSi154* |
| GS10032 | *arSi155* |
| GS10033 | *arSi156* |
| GS10060 | *arSi164* |
| GS10065 | *arSi169* |
| GS10079 | *arSi174* |
| GS10080 | *arSi175* |
| GS10089 | *arSi183* |
| GS10113 | *arSi194* |
| GS10115 | *arSi196* |
| GS10124 | *arSi155; arTi145; nre-1(hd20) lin-15B(hd126)* |
| GS10126 | *arTi481; lit-1(ar665); arIs51; arTi443; arTi237* |
| GS10127 | *arSi155; lit-1(ar665); arTi483; arTi443; arTi237* |
| GS10128 | *hlh-2(ar623); lit-1(ar665); arTi483; arTi443; arTi237* |
| GS10129 | *pop-1(he335); lit-1(ar665); arTi483; arTi443; arTi237* |
| GS10133 | *nhr-67(syb509ar667); arTi112* |
| GS10134 | *hlh-2(ar623ar668); arTi112* |
| GS10147 | *nhr-67(syb509); arTi112* |
| GS10157 | *hlh-2(ar623); arTi112; nhr-67(pf88)* |
| GS10160 | *arSi214; arTi145;nre-1(hd20) lin-15B(hd126)* |
| GS10179 | *arSi169; lit-1(ar665); arTi483; arTi443; arTi237* |
| GS10180 | *hlh-2(ar657); nhr-67(syb509ar667)* |
| GS10181 | *hlh-2(ar657); nhr-67(syb509)* |
| GS10182 | *arSi155; lsy-12[ot170]* |
| GS10183 | *arSi155; lsy-12[ot171]* |
