# Supplementary figures and images for: Regulation of hlh-2 transcription during specification of the anchor cell of the C. elegans hermaphrodite gonad
Source: G3 (Bethesda). 2025 Dec 2;16(2):jkaf288. doi: 10.1093/g3journal/jkaf288 (PMC12869078; doi:10.1093/g3journal/jkaf288)

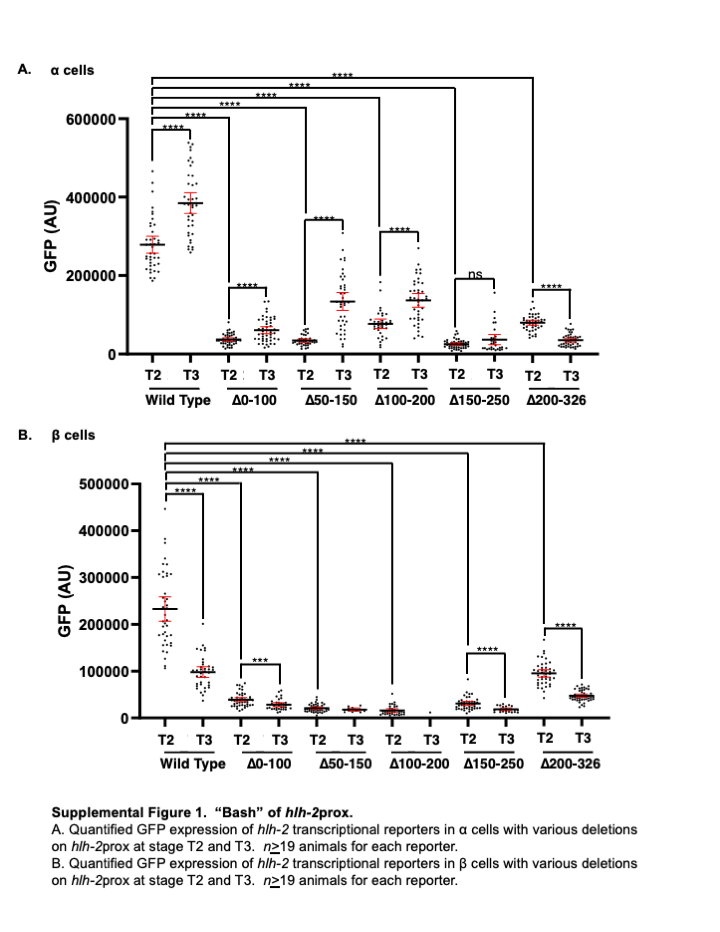

Supplement: jkaf288_Supplementary_Data [file jkaf288_supplementary_data.zip › Supplemental_Figure_1_G3-2025-406240.tif]

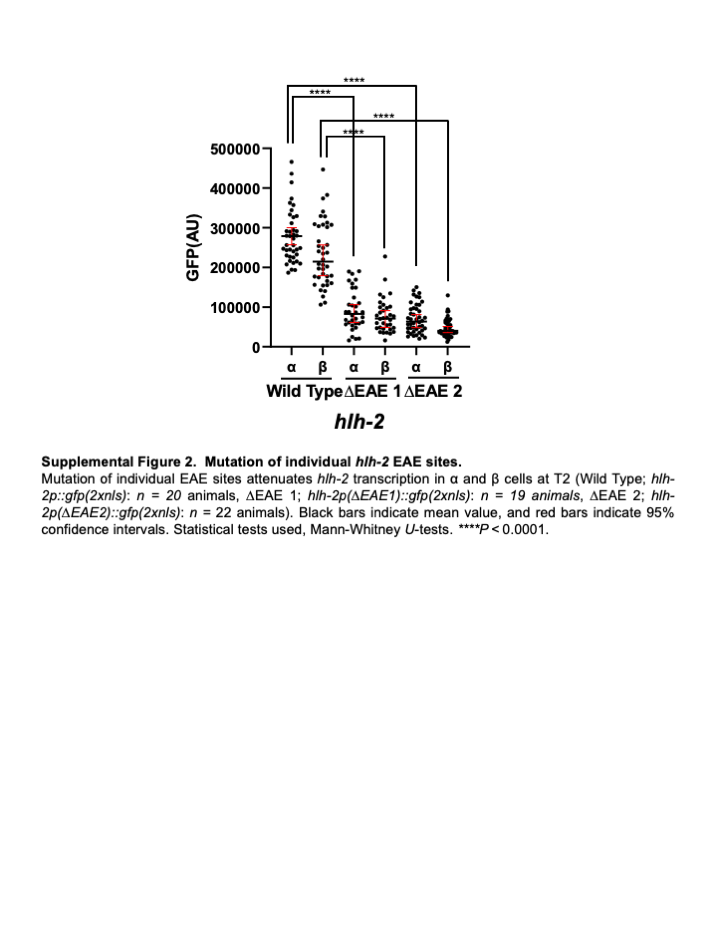

Supplement: jkaf288_Supplementary_Data [file jkaf288_supplementary_data.zip › Supplemental_Figure_2_G3-2025-406240.tif]

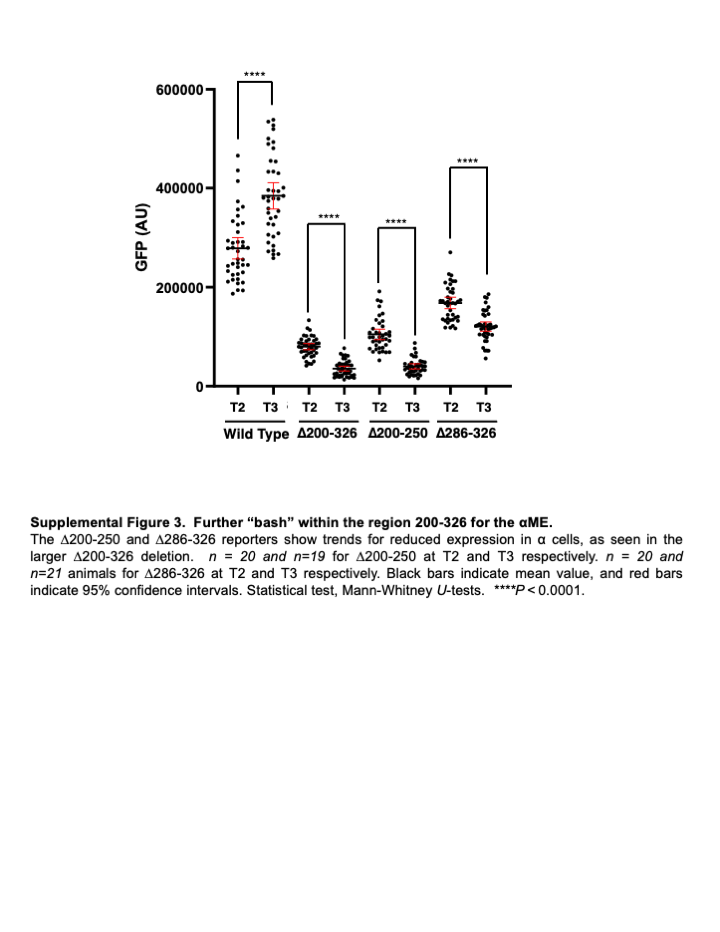

Supplement: jkaf288_Supplementary_Data [file jkaf288_supplementary_data.zip › Supplemental_Figure_3_G3-2025-406240.tif]
